# Effect of E-Beam Irradiation on Solutions of Fullerene C_60_ Conjugate with Polyvinylpyrrolidone and Folic Acid

**DOI:** 10.3390/polym17091259

**Published:** 2025-05-05

**Authors:** Anna V. Titova, Zhanna B. Lyutova, Alexandr V. Arutyunyan, Aleksandr S. Aglikov, Mikhail V. Zhukov, Lyudmila V. Necheukhina, Darya V. Zvyagina, Victor P. Sedov, Maria A. Markova, Anton V. Popugaev, Alina A. Borisenkova

**Affiliations:** 1 Radiation Technology Department, St. Petersburg State Institute of Technology, Technical University, 190013 St. Petersburg, Russia; 2 Petersburg Nuclear Physics Institute Named by B.P. Konstantinov of National Research Centre “Kurchatov Institute”, 188300 Gatchina, Russia; 3 Infochemistry Scientific Center, ITMO University, 191002 St. Petersburg, Russia; 4 Institute for Analytical Instrumentation of the Russian Academy of Sciences, 198095 St. Petersburg, Russia; 5 School of Physics and Engineering, ITMO University, 197101 St. Petersburg, Russia

**Keywords:** poly(N-vinyl pyrrolidone), fullerene, E-Beam irradiation, radiation-induced cross-linking, intermolecular cross-linking, intrinsic viscosity

## Abstract

The radiation sterilization of polymer-based drug solutions can change the characteristics that determine the efficiency of drug targeting, such as particle sizes in the solution and their surface potential. The effect of E-beam treatment at doses of 3 and 8 kGy in a Xe or air atmosphere on the hydrodynamic properties of dilute solutions of polyvinylpyrrolidone (PVP) conjugate with fullerene C_60_ and folic acid (FA-PVP-C_60_) was studied and compared with native PVP K30. The capillary viscometry method was used to determine the intrinsic viscosity of solutions. The particle sizes (*R_h_*) were determined using the DLS method. The zeta potential of the particles was determined using the PALS method. The morphological features of the conjugate surface irradiated in a Xe atmosphere with a dose of 8 kGy FA-PVP-C_60_ were studied by AFM. The functionalization of FA-PVP-C_60_ and PVP during E-beam treatment was examined using UV- and FTIR-spectrometry. When the diluted solutions of FA-PVP-C_60_ and PVP were irradiated in air with a dose of 3 kGy, destruction of polymer chains occurred predominantly, but when the dose was increased to 8 kGy, intermolecular cross-linking occurred, leading to an increase in the characteristic viscosity and particle size in the solution. It was shown that the average particle sizes, amounting to 3 and 8 nm for PVP and 4 and 20 nm for FA-PVP-C_60_, did not change significantly under E-beam irradiation in a Xe atmosphere in the considered dose range. The zeta potential of the particles remained virtually unchanged for both PVP and FA-PVP-C_60_ under all irradiation conditions. The obtained results indicate the possibility of performing radiation sterilization of FA-PVP-C_60_ conjugate solutions in an inert gas atmosphere in the range of studied doses.

## 1. Introduction

An important parameter of drugs is their bioavailability [1]. The poor solubility of drugs leads to a decrease in their therapeutic effect, while an increase in the content of the active substance can lead to an increase in toxicity [2]. Polyvinylpyrrolidone (PVP) is a promising polymer drug carrier due to its hydrophilic properties [3]. At the same time, PVP prevents the aggregation of nanoparticles due to the steric effect stemming from the repulsion of its hydrophobic chains [4]. An important property of PVP is the presence of pyrrolidone, which can strongly bind to nanoparticles via chemisorption [5]. These properties of PVP contribute to the creation of water-soluble complexes with hydrophobic carbon nanoparticles, including fullerenes [6,7,8,9,10,11]. We previously obtained a conjugate of fullerene C_60_ with folic acid (FA) and PVP as a prototype of a radioisotope carrier for targeted delivery to tumor cells with overexpressed folate receptors [12]. The PVP in the FA-PVP-C_60_ conjugate was used as a biocompatible hydrophilic spacer, improving the bioavailability of the poorly water-soluble fullerene and FA. However, the creation of such a conjugate based on endohedral metallofullerene containing a radioactive isotope requires an understanding of the effect of ionizing radiation on the hydrodynamic properties of PVP-based conjugate solutions, as their radical change may complicate the use of such compounds in vivo.

It is known that the processes occurring in PVP solutions under the influence of ionizing radiation depend on parameters such as the type of radiation [13], dose and dose rate [14], irradiation atmosphere [15,16], temperature [17], polymer molecular weight [13], and concentration of the solution. The effect of ionizing radiation with a high dose rate on dilute PVP solutions leads to the formation of nanogels due to the predominance of the intramolecular cross-linking process [14,18,19,20], while the effect of ionizing radiation with a low dose rate on concentrated PVP solutions leads to the formation of macrogels and wall-to-wall gels due to the predominance of intermolecular cross-linking [21]. From the point of view of biomedical applications, the formation of nanogels is preferable, as their characteristic size (up to 100 nm) allows them to penetrate into cells. In addition, under the action of radiation, the PVP molecule can be modified with the functional groups either generated as a result of intramolecular radical reactions or specially grafted via radiation copolymerization [22,23,24]. This allows for the conjugation of therapeutic drugs, making PVP-based nanogels suitable carriers for biomedical applications [22,25,26,27,28,29]. Another notable advantage of nanogel synthesis via the radiation method is that, depending on the radiation dose used, sterilization of the drug can occur simultaneously [30]. Sterilization by ionizing radiation offers a number of advantages over other methods. These include an unrivaled efficiency in inactivating microorganisms, the processing of products in final transport packaging, and the removal of the need for toxic chemicals (e.g., ethylene oxide) and high temperatures. At the same time, the main fundamental issue of the radiation sterilization of drugs is determining a dose sufficient for reliable sterilization [31] that does not lead to a change in chemical, hydrodynamic, and biological properties that disrupts the functionality of biomaterials [30,32]. While previous investigations have mainly studied changes in native PVP under the influence of irradiation, this work is the first to examine the effect of E-beam treatment on the already prepared FA-PVP-C_60_ conjugate.

The aim of this work was to study the effect of E-beam radiation on the hydrodynamic properties of PVP conjugate solutions with fullerene C_60_ and FA (FA-PVP-C_60_) and to compare them with native PVP. Considering that the sterilization dose can reach 2 kGy [33], we limited the radiation dose up to 8 kGy in our study. In addition, the use of a high dose may result in the radiolysis of FA in the conjugate under the influence of radiation [34,35].

## 2. Materials and Methods

### 2.1. Materials

Poly(N-vinylpyrrolidone) (PVP K30) with a molecular weight of 40.0 kDa (according to the manufacturer’s information) was purchased from Sisco Research Laboratories (Pvt. Ltd., Maharashtra, India). The FA-PVP-C_60_ conjugate was prepared as described previously [12]. The solutions of PVP K30 and FA-PVP-C_60_ with a concentration of 0.5 g/dL were prepared in double-deionized water by stirring at 500 rpm for 1 h at room temperature. Before irradiation, the solutions were passed through Minisart syringe filters (Sartorius, Gottingen, Germany), first with a pore size of 0.45 μm and then a pore size of 0.2 μm.

### 2.2. Methods

#### 2.2.1. E-Beam Irradiation

The 0.5 g/dL (pH = 7) solutions of PVP K30 and FA-PVP-C_60_ were poured into hermetically sealed 15 mL glass vials and deoxygenated by blowing xenon for 1 h. Similar solutions equilibrated with air were also prepared. Irradiation was performed with the high-energy electrons accelerated to 10 MeV on a Mevex MB10-30SC900 linear accelerator (STERIS AST Equipment and Technologies, Mentor, OH, USA). Irradiation doses were 3 and 8 kGy (dose rate of 0.1 kGy/s) at room temperature. Dose control was carried out using a film dosimeter CO(PDE) 1-10 (VNIIFTRI, Zelenograd, Russia).

#### 2.2.2. Capillary Viscometry

Viscosity measurements were performed using a VPZH-2 glass capillary viscometer (EKROSKHIM LCC, St. Petersburg, Russia). The accuracy of this type of viscometer depends on its design; therefore, the liquid flow time does not depend on the hydrostatic pressure or the amount of liquid. The viscometer constant was 0.009059 mm^2^ s^−2^. The temperature of the studied samples was maintained using a thermostatic bath with an accuracy of 25.00 ± 0.01 °C. The flow time was maintained with an accuracy of ±0.1 s. The viscometer was thoroughly washed with boiling distilled water, dried, and reinstalled after each experiment. The relative viscosity of the solutions was determined using Equation (1):

(1) $$\eta_{r}=\frac{\tau }{\tau_{0}}$$

where *τ* is the solution flow time, measured in s, and *τ*_0_ is the solvent flow time, also measured in s.

#### 2.2.3. Dynamic Light Scattering and Zeta Potential Measurements

Experiments to determine the particle sizes in the PVP and FA-PVP-C_60_ conjugate solutions at various concentrations were performed via dynamic light scattering (DLS) at 25 °C using a Photocor Compact-Z analyzer (Photocor LLC, Moscow, Russia) with a laser wavelength of 637.7 nm and a maximum light beam power of 25 mW at a scattering angle of 90°. The distributions of the translational diffusion coefficients, *D_T_*, of light-scattering particles were obtained by analyzing the autocorrelation function of the intensity of light scattered by the samples using DynaLS software (Vers. 2.9.1, Dr. Alexander Goldin, Alango Ltd., Tirat Carmel, Israel). Measurements were performed at several solution concentrations to determine the diffusion coefficient *D*_0_ at the ultimate dilution. The hydrodynamic radius of *R_h_* particles in the solution was calculated according to the Stokes–Einstein relation using Equation (2), as follows:

(2) $$R_{h}=\frac{kT}{6\pi \eta_{0}D_{0}}$$

where *k* is the Boltzmann constant, *T* is the absolute temperature, and *η*_o_ is the viscosity of the liquid.

Zeta potential measurements using laser Doppler anemometry were performed on the same analyzer. Doppler shift analysis of the studied samples was performed using the PALS (Phase-Analysis Light Scattering) method at 25 °C. The stability of the particle size distribution and zeta potential was determined based on at least three measurements of each sample.

#### 2.2.4. Atomic Force Microscopy

AFM images of the unirradiated and irradiated samples cast on silicon substrates were obtained in contact mode using the NTEGRA-Aura (NT-MDT LLC, Moscow, Zelenograd, Russia). A high-resolution silicon probe (Nsg01 from NT-MDT) with a nominal spring constant of 5 N∙m^−1^ and a resonant frequency of 150 kHz was used.

#### 2.2.5. IR-Fourier Spectrometry

IR-Fourier spectrometry was performed on an IRTracer-100 spectrometer (Shimadzu, Kyoto, Japan). The spectra were recorded at 32 scans per spectrum and a resolution of 4 cm^−1^ in the range of 4000–400 cm^−1^. The analyzed substances dispersed with potassium bromide were pressed into pellets.

#### 2.2.6. UV-Vis Spectrometry

The absorption spectra of PVP and FA-PVP-C_60_ in water were obtained using UV-Vis spectrometry on an SF-2000 spectrophotometer (OKB Spectr LLC, St. Petersburg, Russia).

## 3. Results and Discussion

### 3.1. Intrinsic Viscosity

**Figure 1:**
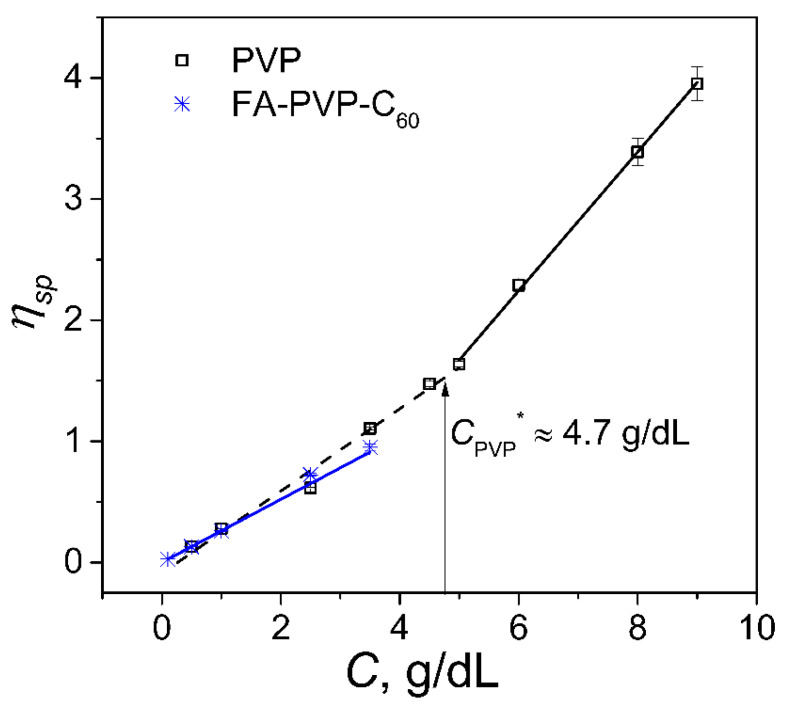
The dependence of the specific viscosity on the concentration of PVP and the FA-PVP-C_60_ conjugate.

In cases where the exact molecular structure of a polymer-based conjugate is unknown, the determination of the intrinsic viscosity allows researchers to establish the main trends in the physical properties of biopolymer systems [36]. Drugs with high viscosity require the optimization of the injection process [37]. Therefore, we evaluated the change in the intrinsic viscosity of PVP and the FA-PVP-C_60_ conjugate during irradiation under different conditions. Figure 1 shows that the change in specific viscosity depends on the concentration of PVP and FA-PVP-C_60_. For PVP, the change in the slope of this dependence at a certain concentration, C*, is obvious. This value, equal to 4.75 g/dL for PVP K30, is called the overlap concentration and corresponds to the previously obtained value [38]. It is believed that below C*, there is a region of dilute solutions, and polymer coils that do not overlap with each other can be considered individual molecules [39]. The solubility in water of the FA-PVP-C_60_ conjugate containing poorly water-soluble components—fullerene and FA—is significantly lower compared with the native PVP K30. For the FA-PVP-C_60_ conjugate, we did not observe a change in the slope of the specific viscosity dependence similar to that observed in PVP, perhaps because the overlap concentration is not achieved at the maximum possible solubility in water of the FA-PVP-C_60_ conjugate. The probability of the intermolecular cross-linking of PVP chains is high when the irradiating solution’s concentration exceeds the overlap concentration. Intermolecular cross-linking can lead to a significant increase in the intrinsic viscosity, molecular weight, and particle size of the polymer, which can have an extremely negative effect on the possibility of using such a drug in biomedicine. Therefore, for irradiation, we selected a concentration of 0.5 g/dL, which was almost an order of magnitude less than the PVP overlap concentration.

To determine the intrinsic viscosity [*η*] of the unirradiated and irradiated solutions of PVP and FA-PVP-C_60_, the Huggins (3) [40] and Kraemer (4) [41] equations were used:

(3) $$\frac{\eta_{sp}}{c}=\eta +k^{\prime }\eta^{2}c+\cdots$$

where *η_sp_* = *η_r_*−1, and *k*′ is the Huggins coefficient, which characterizes the interaction of the flexible-chain polymer with the solvent.

(4) $$\frac{ln\eta_{r}}{c}=\eta +k^{\prime \prime }\eta^{2}c+\cdots$$

where *k*″ is the Kraemer coefficient.

In the case of discrepancy between the values obtained through the extrapolation of the Huggins and Kremer graphs to zero concentration, the intrinsic viscosity was determined as the average between the two extrapolated values. The data obtained are presented in Table 1.

Using the obtained values of characteristic viscosity, the viscosity average molecular weight of unirradiated PVP was calculated according to the Mark–Houwink [42] equation as follows:

(5) $${\bar{M}}_{v}=8.04\;\cdot \;10^{5}{\left[\eta \right]}^{1.82}$$

The value of *M_v_* obtained for PVP (Table 1) is in good agreement with the value of the average molecular weight *M_w_* (51 kDa), which was obtained by us using the Debye method to process the light-scattering data. We did not determine the value of the viscosity average molecular weight of the FA-PVP-C_60_ conjugate, since the coefficients in the Mark–Houwink equation are unknown in this case. In addition, the decrease in the intrinsic viscosity of the irradiated samples is not necessarily associated with a decrease in the length of the polymer chain but may be a consequence of a decrease in the size of the polymer coils due to intramolecular cross-linking. At the same time, an increase in the intrinsic viscosity of the irradiated samples most likely indicates the predominance of intermolecular cross-linking processes. During irradiation in air, at a dose of 3 kGy, a slight decrease in the intrinsic viscosity of both PVP and the FA-PVP-C_60_ conjugate occurred. In this case, it is most likely that the destruction of polymer chains occurs due to the reaction of molecular oxygen with polymer macroradicals, leading to the formation of peroxyl radicals, followed by the β-scission of oxyl radicals [15]. However, as noted by Ditta et al. [15], this reaction competes with combination/disproportionation [43] only in the early stage of irradiation. The oxygen in the solution is quickly consumed during irradiation, following which irradiation occurs in an inert atmosphere. Afterward, as can be seen in Table 1, with an increase in the dose to 8 kGy during irradiation in air, an increase in intrinsic viscosity occurs, which is probably a consequence of the increase in the molecular weight of both PVP and the FA-PVP-C_60_ conjugate. It is evident that the number of radicals on the PVP chains shortened by the low doses of irradiation is so small that the probability of intramolecular cross-linking is significantly lower compared to intermolecular cross-linking.

In the case of irradiation in an inert gas atmosphere (xenon), we observed a dose-dependent decrease in the characteristic viscosity of both PVP and the FA-PVP-C_60_ conjugate (Table 1). In this case, it is most likely that the intramolecular cross-linking of polymer molecules predominantly occurs, leading to a decrease in their size; therefore, we did not evaluate the viscosity-average molar mass. The formation of internally cross-linked nanogels upon the irradiation of diluted PVP K90 solutions in an inert gas atmosphere was previously reported by T. Balogh et al. [16].

### 3.2. Hydrodynamic Radius and Zeta Potential

The data obtained on the basis of the change in the characteristic viscosity, indicating differences in the processes occurring during the irradiation of PVP and the FA-PVP-C_60_ conjugate, require confirmation by other methods. Traditionally, the formation of nanogels during PVP irradiation is determined by the decrease in the radius of gyration of the polymer coil with a constant molecular weight. However, we did not set ourselves to the task of synthesizing nanogels, the production of which requires higher molecular PVP and irradiation in a N_2_O atmosphere in order to increase the radiation-chemical yield of hydroxyl radicals [16]. It is known that the size of the particles in a solution can affect their cytotoxicity and play an important role in their interaction with biological objects and the possibility of penetrating through the cell membrane. We needed confirmation that the particle size of the FA-PVP-C_60_ conjugate subjected to radiation sterilization in solution still corresponded to the optimal size for biomedical applications [44]. Therefore, we opted to determine the hydrodynamic radius of the particles in solution using DLS.

**Figure 2:**
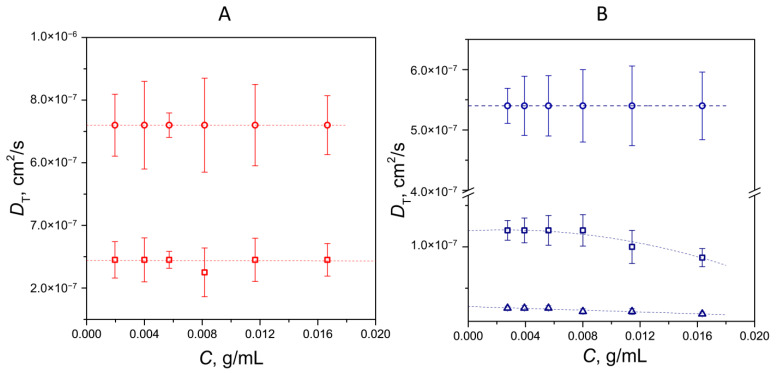
The dependence of the translation diffusion coefficient of the PVP (**A**) and the FA-PVP-C_60_ conjugate (**B**) vs. the solution’s concentration.

Diffusion in a sufficiently concentrated polymer solution may be due to the viscosity of the solution itself rather than that of the solvent. In this case, the true diffusion coefficient (and hence the true particle size) can be obtained at a limit of small concentration values when the diffusion coefficient itself ceases to depend on the concentration. Figure 2 shows the plots of the translational diffusion coefficient versus concentration for PVP (Figure 2A) and the FA-PVP-C_60_ conjugate (Figure 2B).

PVP exhibits a bimodal size distribution that is virtually independent of concentration (Figure 2A). The measurements of aqueous PVP solutions revealed the presence of two “fast” modes of translational diffusion coefficients associated with the presence of freely moving particles, which may correspond to individual PVP chains or small aggregates of several chains. Extrapolation of these modes to zero concentration corresponds to hydrodynamic radii of 3 and 8 nm. The FA-PVP-C_60_ conjugate exhibits a trimodal size distribution (Figure 2B). The fast mode, corresponding to a larger diffusion coefficient, is also independent of concentration and corresponds to a hydrodynamic radius of 4.5 nm. The two slower modes, exhibiting a slight concentration dependence, yield hydrodynamic radii of 20 and 88 nm. Due to the presence of poorly water-soluble components, such as fullerene C_60_ and folic acid, in the FA-PVP-C_60_ conjugate, it can be assumed that the slow modes occur because of the formation of aggregates due to the hydrophobic interactions of the conjugates.

**Figure 3:**
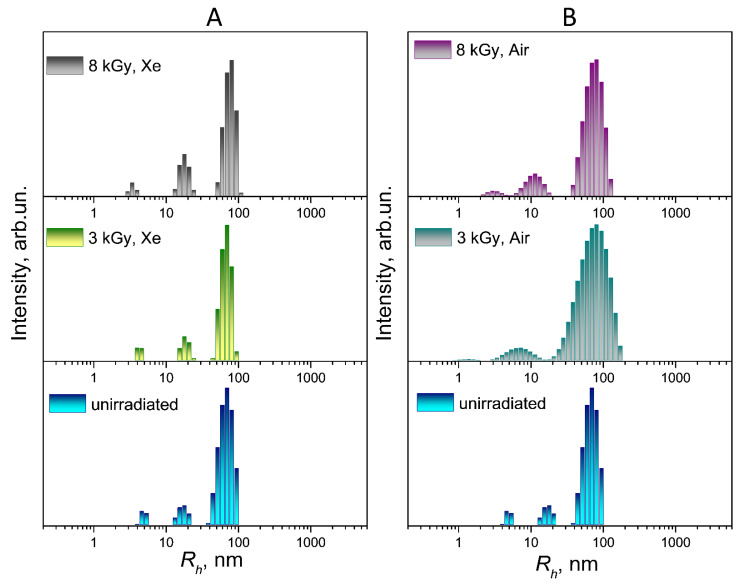
Distribution of particle sizes by light scattering intensity of FA-PVP-C_60_ (*c* = 0.5 g/dL) irradiated with doses of 3 and 8 kGy in xenon (**A**) or air (**B**) atmosphere.

**Figure 4:**
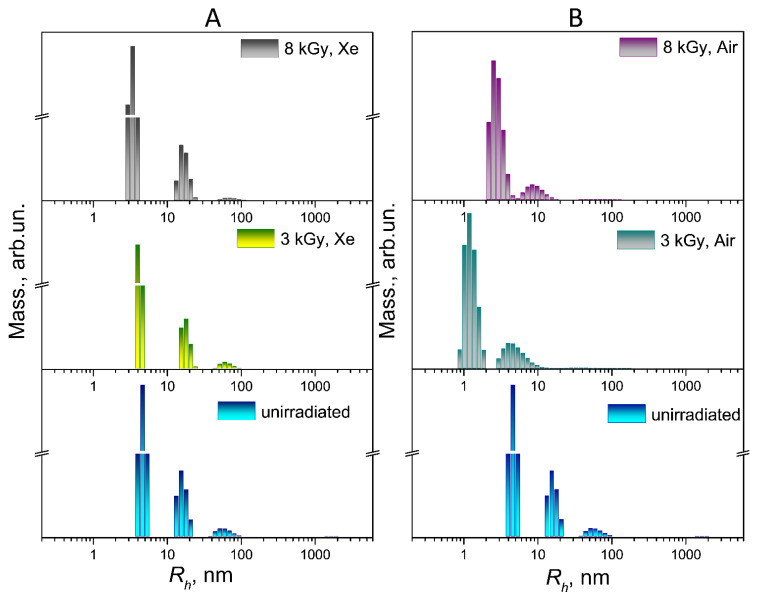
Distribution of particle sizes by mass of FA-PVP-C_60_ (*c* = 0.5 g/dL) irradiated with doses of 3 and 8 kGy in xenon (**A**) or air (**B**) atmosphere.

A comparison of the particle size distributions of the unirradiated and irradiated FA-PVP-C_60_ conjugate solutions (*c* = 0.5 g/dL) under various conditions by light scattering intensity and mass is shown in Figure 3 and Figure 4, respectively.

When the conjugate was irradiated in a xenon atmosphere, with an increase in the irradiation dose, a slight shift towards smaller formations was observed in the fraction corresponding to the smallest particle size in the size distributions by light scattering intensity and by mass (Figure 3A and Figure 4A). The observed change in the hydrodynamic radius of the conjugate particles during irradiation may indicate the formation of internally cross-linked compact polymer coils. This is consistent with the decrease in the characteristic viscosity of the conjugate as the irradiation dose increases in a xenon atmosphere.

In the case of irradiation of the FA-PVP-C_60_ conjugate solution in air with a dose of 3 kGy (Figure 3B), a significant broadening of the intensity distribution of the largest fraction and a shift in the middle and smaller fractions towards smaller values were noted. This shift, as well as the broadening of the distribution, may indicate an increase in the polydispersity of the sample due to the prevalence of the carbon chain destruction processes. At a dose of 8 kGy, we did not observe a significant increase in particle size, expected due to the increase in the intrinsic viscosity of the conjugate solution irradiated under these conditions.

Nanoconjugates intended for targeted drug delivery must have a certain functionality; they must penetrate a specific cell, not be eliminated by the reticuloendothelial system, and circulate in the blood for the time required for the expected therapeutic effect to occur [45]. Therefore, a slight change in the size of the FA-PVP-C_60_ particles under the influence of irradiation in a xenon atmosphere should not have a significant effect on the rate of elimination of conjugates from the bloodstream. A nanoparticle can use several routes to penetrate the cell, depending on its size. The optimal size (diameter) of particles for cellular uptake is 40–60 nm [46]. In addition, 30–50 nm nanoparticles effectively interact with receptors and are subsequently internalized by the cell via receptor-mediated endocytosis [47]. Both unirradiated FA-PVP-C_60_ conjugates and those irradiated in xenon contain particles of an optimal size (*R_h_*~20 nm) for cellular uptake (Figure 4A). At the same time, the prevalence of destruction processes during the irradiation of air-equilibrated conjugate solutions with low doses can lead to a loss of the conjugate-targeting ability due to the polymer chain scission involved in conjugation with the FA. Therefore, in order to preserve the functionality of the conjugate, irradiation in an inert gas atmosphere is preferable.

**Figure 5:**
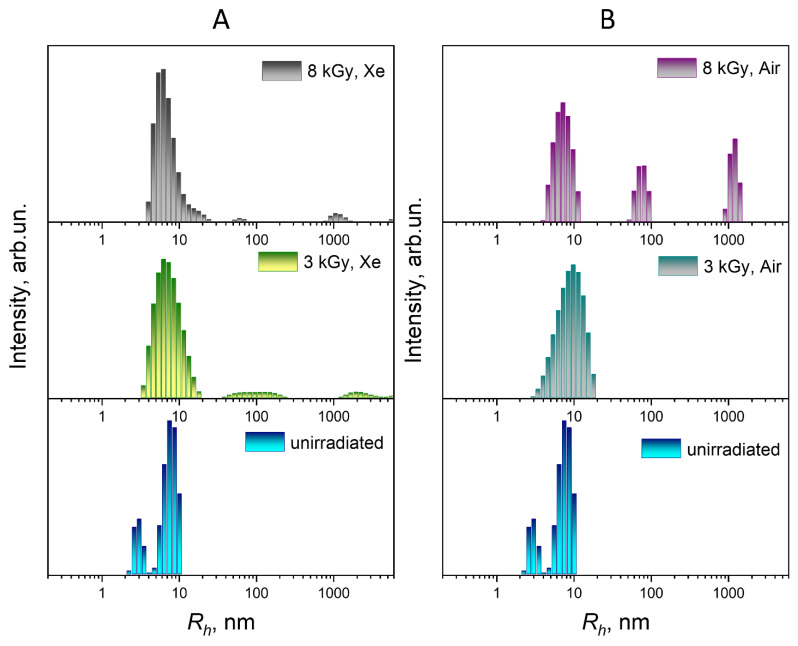
Distribution of particle sizes by light scattering intensity of PVP (*c* = 0.5 g/dL) irradiated with doses of 3 and 8 kGy in xenon (**A**) or air (**B**) atmosphere.

**Figure 6:**
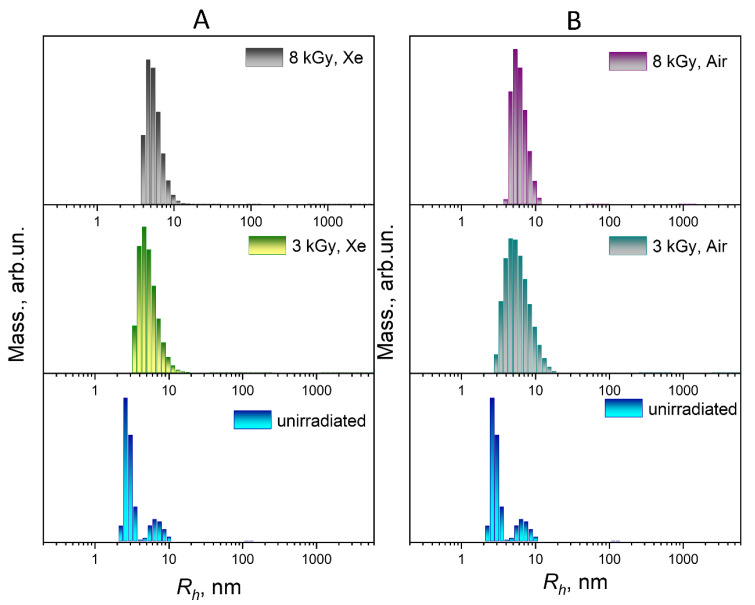
Distribution of particle sizes by mass of PVP (*c* = 0.5 g/dL) irradiated with doses of 3 and 8 kGy in xenon (**A**) or air (**B**) atmosphere.

In the particle size distribution by the light scattering intensity of native PVP particles (Figure 5A) irradiated with a dose of 3 kGy in a xenon atmosphere, a small number of micron-sized aggregates appear, which may indicate the simultaneous occurrence of both intramolecular and intermolecular cross-linking processes. In the particle size distribution by the mass of PVP particles irradiated in a xenon atmosphere (Figure 6A), a dose-dependent shift towards smaller sizes was not observed for either fraction. The particle size distribution of PVP irradiated with a dose of 8 kGy becomes narrower in both intensity (Figure 5A) and mass representation (Figure 6A), which may indicate intramolecular cross-linking. The particle size distribution of PVP irradiated with a dose of 8 kGy in a xenon atmosphere becomes slightly narrower in both intensity (Figure 5A) and mass representation (Figure 6A), which may indicate intramolecular cross-linking.

When PVP solutions were irradiated at a dose of 3 kGy in air, as in the case of the conjugate, a broadening of light scattering intensity distribution was observed (Figure 5B), which may indicate the prevalence of the polymer chain destruction process. When irradiated with a dose of 8 kGy, large aggregates reaching micron size appear, but their contribution to the mass representation of the size distribution is extremely small (Figure 6B).

**Figure 7:**
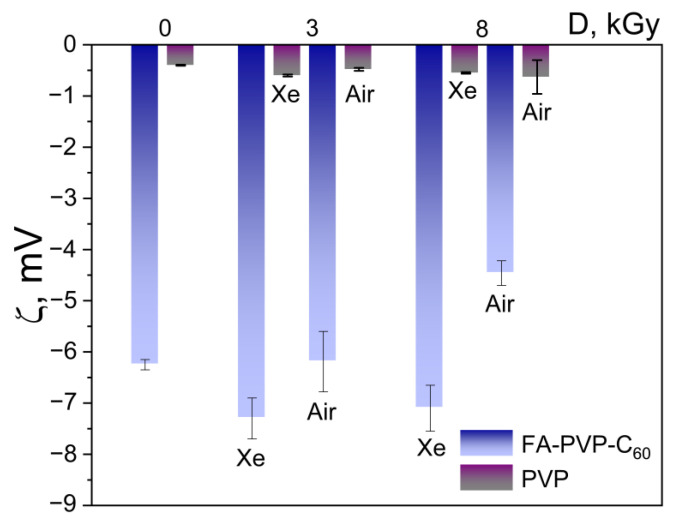
Zeta potential ± SD of FA-PVP-C_60_ and PVP measured by PALS.

An important parameter characterizing the stability of colloidal systems is the zeta potential. It was observed that the absolute value of the zeta potential of the conjugate is higher than that of the practically neutral PVP (Figure 7). No significant change in the zeta potential was observed in both the FA-PVP-C_60_ conjugate and native PVP samples irradiated under different conditions. This may indicate the relative stability of these solutions to the action of E-beam treatment, at least at the used irradiation doses.

### 3.3. PVP and FA-PVP-C_60_ Functionalization Under Irradiation

**Figure 8:**
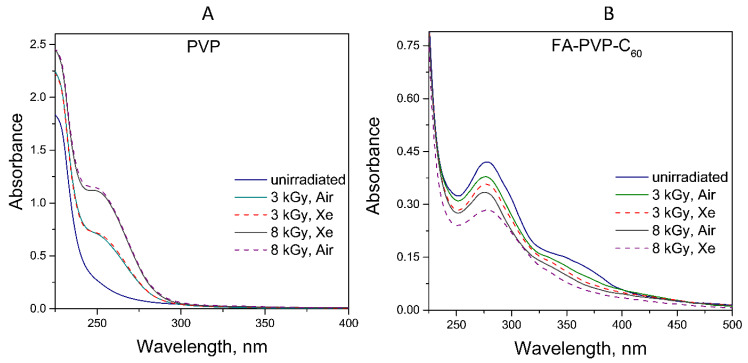
UV-Vis spectra of the PVP (*c* = 5mg/mL) (**A**) and FA-PVP-C_60_ (*c* = 200 µg/mL) conjugate (**B**) solutions irradiated under various conditions.

PVP can undergo functionalization under the influence of ionizing radiation [15,48]. The formation of radical adducts of PVP-OH and enol has been proposed as a possible intramolecular reaction [17]. Figure 8 shows the absorption spectra of the PVP and FA-PVP-C_60_ conjugate solutions subjected to E-beam irradiation under different conditions. It was found that a peak in the 250 nm region appears in the absorption spectrum of PVP (Figure 8A), the intensity of which increases with increasing dose. In this case, the absorption value did not depend on the irradiation atmosphere. This result indicates the formation of an unsaturated functional group in PVP, probably evolving towards the formation of an enol structure [17]. In the UV spectrum of the FA-PVP-C_60_ conjugate, it is difficult to identify the maximum in this region since this region contains the maximum absorption of FA [12,49]. Under the influence of E-beam irradiation, the radiolysis of FA in the conjugate occurs, leading to the dose-dependent decrease in absorption in the characteristic maxima of FA [34,35]. At the same time, about 85% of FA remains undestroyed in the conjugate after irradiation with a dose of 3 kGy.

**Figure 9:**
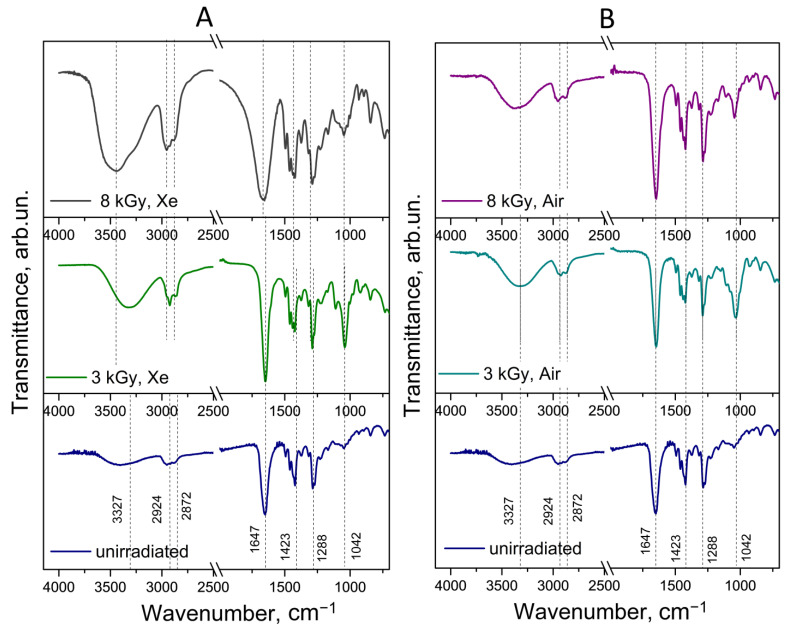
FTIR spectra of the FA-PVP-C_60_ conjugate irradiated in a xenon (**A**) or air (**B**) atmosphere in comparison with the unirradiated conjugate.

**Figure 10:**
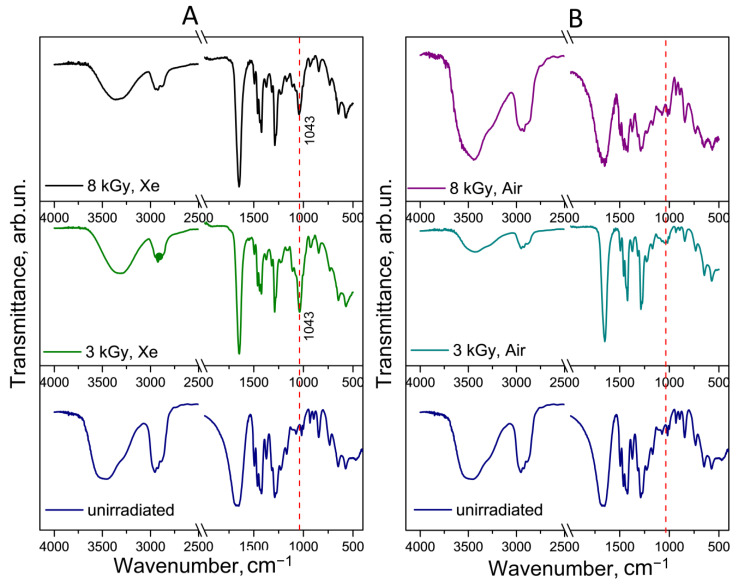
FTIR spectra of the PVP conjugate irradiated in a xenon (**A**) or air (**B**) atmosphere in comparison with the unirradiated PVP. The red dotted line corresponds to the stretching of the C–OH bond.

The formation of hydroxyl derivatives in PVP should be accompanied by the appearance of corresponding bands in the FTIR spectrum. Figure 9 and Figure 10 show the FTIR spectra of the FA-PVP-C_60_ conjugate and PVP irradiated under various conditions in comparison with the unirradiated samples. The FTIR spectra of the unirradiated FA-PVP-C_60_ conjugate contain all the main bands characteristic of PVP. Thus, a broad band was observed in the region of 3327 cm^−1^, responsible for the ν(O–H) vibrations, as well as bands in the region of 2920–2870 cm^−1^, responsible for the ν(–CH_2_) vibrations and asymmetric ν(C–H) vibrations. The strong band at 1651 cm^−1^ is responsible for the ν(C=O) vibrations, the bands in the region of 1400 cm^−1^ are responsible for the scissor and symmetric vibrations of ν(–CH_2_), the band at 1228 cm^−1^ is responsible for the ν(C–N) vibrations, and the band at 1024 cm^−1^ corresponds to the ν(C–N) vibrations [50]. As we noted earlier, the formation of a non-covalently bound conjugate of PVP with fullerene and FA is indicated by a small shift in the band responsible for the vibrations of the C=O bond.

It was noted that there were no significant changes in the FTIR spectra of the irradiated FA-PVP-C_60_ conjugate samples (Figure 9), except for an increase in the intensity of the transmission band at 1042 cm^−1^, corresponding to the stretching of the C–OH bond under all irradiation conditions [51].

At the same time, an increase in the intensity of this band in PVP occurred only when irradiated in a xenon atmosphere (Figure 10). It is probable that, when irradiated in air, PVP is less prone to intramolecular radical reactions compared to intermolecular cross-links. These data are consistent with the data showing an increase in the intrinsic viscosity of PVP irradiated in air. We did not observe changes in the FTIR spectra indicating the formation of unsaturated bonds, cyclic imides, and carboxyl groups, as was noted when the dilute PVP solutions were irradiated at higher doses [15]. An exception is the broadening of the band at 1647 cm^−1^ on the FTIR spectrum when irradiated at a dose of 8 kGy in a xenon atmosphere, which may be a consequence of cross-linking through the recombination of radicals with the carbon center and disproportionation reactions, resulting in the formation of a variety of states for carbonyl groups [48]. Ditta et al. [15] also noted less pronounced spectral changes in PVP samples irradiated in air. This also indicates a lower probability of intramolecular reactions of PVP when irradiated in an oxygen-containing atmosphere.

### 3.4. FA-PVP-C_60_ Surface Morphology

**Figure 11:**
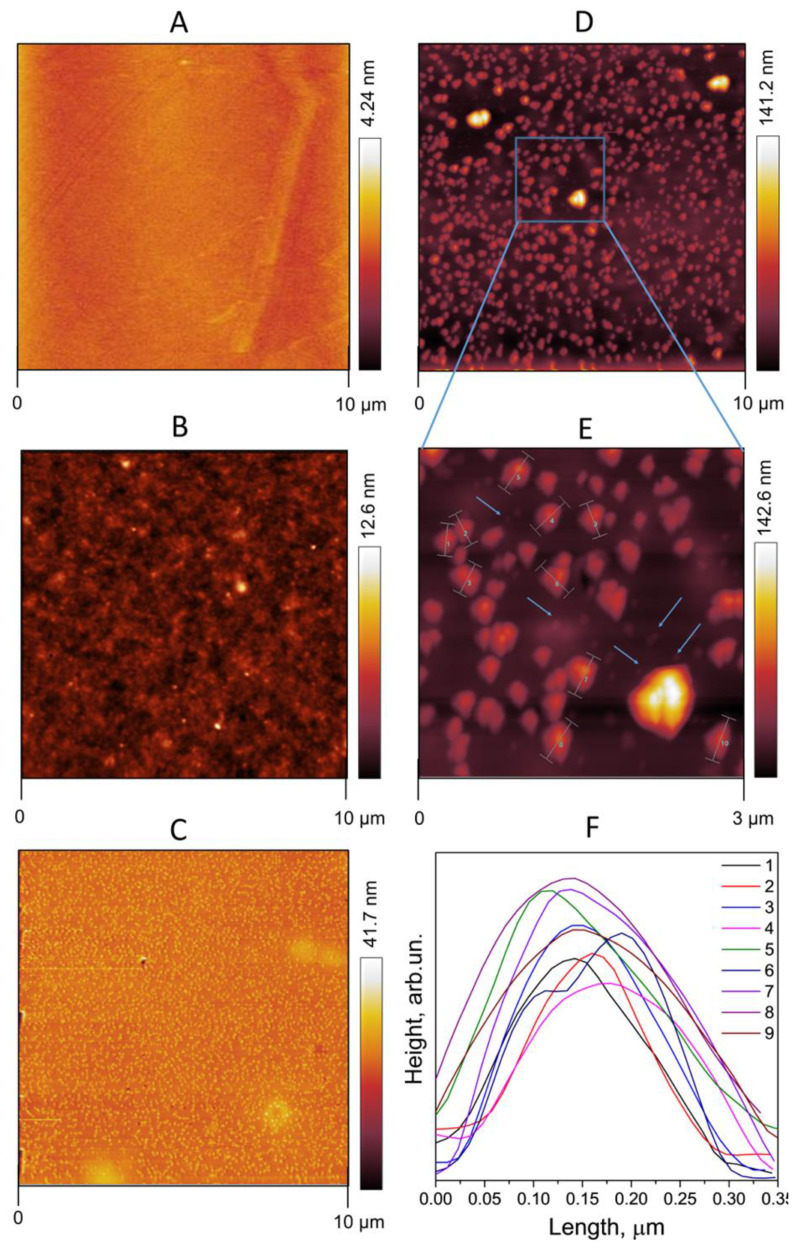
AFM images. (**A**) Pure silicon. (**B**) Dried film of unirradiated PVP. (**C**) Dried film of unirradiated FA-PVP-C_60_. (**D**) Dried film of E-beam-irradiated (8 kGy, xenon) FA-PVP-C_60_. (**E**) Enlarged view of part of (**D**). (**F**) Analysis of particle height profile from previous image.

AFM measurements were used to compare the surface morphology of the unirradiated FA-PVP-C_60_ conjugate and the FA-PVP-C_60_ conjugate irradiated with a dose of 8 kGy in a xenon atmosphere. The unirradiated FA-PVP-C_60_ conjugate (Figure 11C) deposited on a clean silicon substrate (Figure 11A) has a surface profile that is rougher than that of the native PVP (Figure 11B). The image of the irradiated conjugate particles (Figure 11D) shows a completely different characteristic: the particles are even more clearly visible on the surface of the silicon substrate, providing evidence of the presence of cross-linked polymer coils that do not spread over the surface like linear PVP [16,17,52]. At lower magnification (Figure 11D), it seems that the particles of irradiated FA-PVP-C_60_ are spherical in shape and uniform in size, except for a small number of larger aggregates. However, upon zooming in (Figure 11E), it can be seen that medium-sized particles are also formed from smaller particles. As can be seen from the height plot along the particle cross-sections (Figure 11F), indicated by the bars in Figure 11E, their diameter reaches 350 nm. This value is inconsistent with the DLS data, which show that the average particle diameter of the conjugate irradiated with a dose of 8 kGy in a xenon atmosphere is an order of magnitude smaller. It is probable that the large structures are formed from the aggregation of FA-PVP-C_60_ particles during the drying of the film on the silicon surface due to the electrostatic repulsion between the negatively charged conjugate particles [53]. It may also be a consequence of the non-optimal evaporation rate of the solution droplet [54], leading to the appearance of the “coffee ring” effect [55]. However, smaller particles are observed in the AFM image of the irradiated FA-PVP-C_60_ (shown by the arrows in Figure 11E).

**Figure 12:**
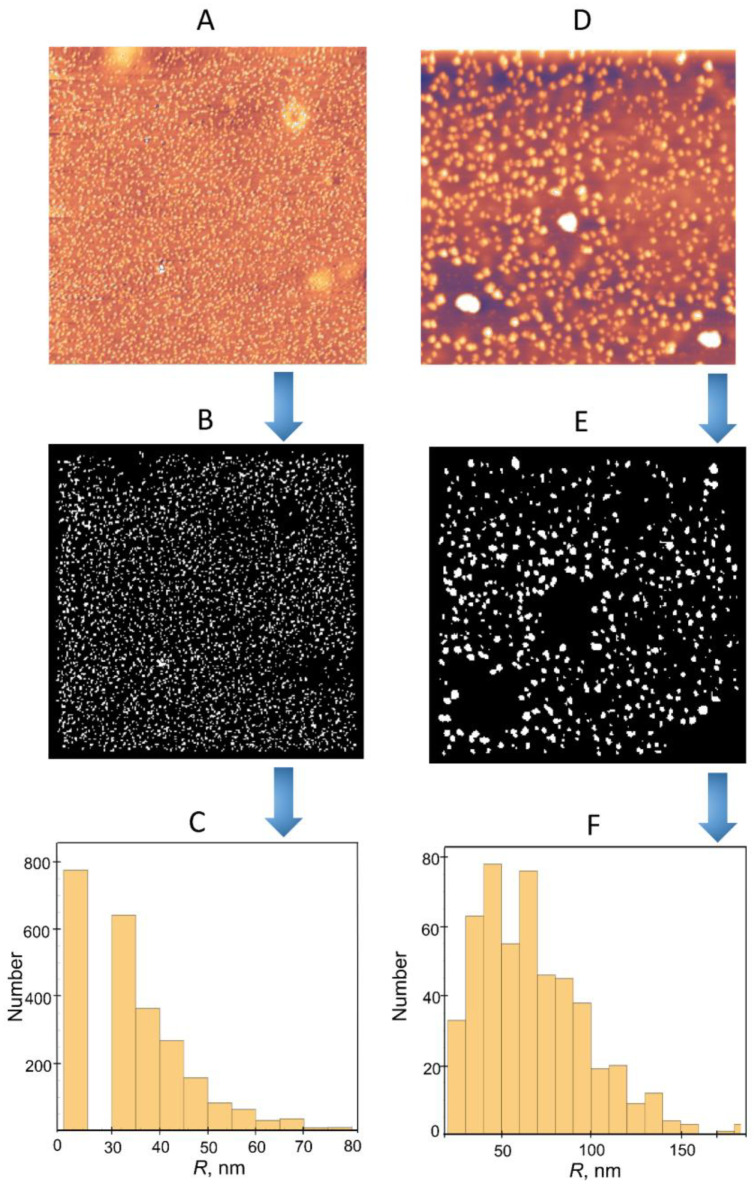
Lateral particle size analysis. (**A**) Topographic AFM image of unirradiated FA-PVP-C_60_. (**B**) Previous image in binarized representation with obvious aggregates and particles at edges excluded. (**C**) Histogram of particle radial distribution of unirradiated FA-PVP-C_60_. (**D**) Topographic AFM image of irradiated (8 kGy, xenon) FA-PVP-C_60_. (**E**) Previous image in binarized representation with obvious aggregates and particles at edges excluded. (**F**) Histogram of particle radial distribution of irradiated FA-PVP-C_60_. Arrows indicate the sequence of lateral particle size analysis.

In addition, an analysis of the distribution of the lateral sizes of unirradiated and irradiated FA-PVP-C_60_ conjugate particles was performed (Figure 12). The topographic AFM images of unirradiated FA-PVP-C_60_ conjugate particles (Figure 12A) and those irradiated at a dose of 8 kGy (Figure 12D) in a xenon atmosphere are shown in Figure 12B,C in a binarized representation. Pixels were converted to units of nm, and particles at the edges, as well as obvious conglomerates, were excluded from the calculation. As can be seen in Figure 12A,C, the histogram of the radius distribution of the irradiated conjugate particles has a larger width compared with the unirradiated conjugate particles. The average values of the radii and their distribution widths are 35.0 ± 15.0 nm and 70.0 ± 40 nm for the unirradiated and irradiated FA-PVP-C_60_ conjugate, respectively. A similar analysis of the particle distribution of PVP nanogel obtained by irradiation was carried out by Sütekin et al. [52]. They also noted the aggregation of nanogel particles during deposition/drying on the mica surface. It should be noted that a difference was also observed between the particle sizes estimated by AFM and DLS methods in irradiated PVP solutions with polyacrylic acid [23].

## 4. Conclusions

This study showed that the processes occurring during the electron irradiation of solutions of both the FA-PVP-C_60_ conjugate and native PVP depend on the irradiation atmosphere. It was found that during the low-dose (3 kGy) irradiation of aqueous solutions of both the FA-PVP-C_60_ conjugate and native PVP equilibrated with air, the polymer chains are destroyed, which was expressed both as a decrease in the intrinsic viscosity and in a decrease in the average particle size. While this may be acceptable for native PVP in some cases, this may lead to a loss of functionality in the case of the FA-PVP-C_60_ conjugate. Radiation destruction of the FA-PVP-C_60_ conjugate may lead to a loss of fullerene or folic acid used as a targeting ligand. Increasing the irradiation dose of air-equilibrated solutions to 8 kGy leads to the formation of intermolecular cross-linked structures. Although the aggregation stability in this case remained virtually unchanged, as evidenced by the virtually unchanged zeta potential value, the increased intrinsic viscosity of the solutions may pose additional challenges during the injection of the drug.

Conversely, the irradiation of both PVP and the conjugate at doses of up to 8 kGy in a xenon atmosphere did not lead to a significant change in hydrodynamic properties, such as the intrinsic viscosity and hydrodynamic radius of particles, as well as the zeta potential. In this case, internally cross-linked polymer structures are formed, which was confirmed by the AFM data.

The irradiation of both PVP and the FA-PVP-C_60_ conjugate led to the formation of new reactive functional groups—hydroxyl derivatives, including those with unsaturated bonds. On one hand, these new groups can be further used for additional conjugation with the necessary molecules to enhance the therapeutic or targeting properties of the conjugate. On the other hand, more biological studies are required to confirm that the appearance of new functional groups does not lead to the toxicity of the polymer used in the FA-PVP-C_60_ conjugate as a biocompatible spacer.

This work shows that in the case of a moderate microbial load, when high doses of radiation are not required, solutions of the FA-PVP-C_60_ conjugate can be subjected to radiation sterilization in an inert atmosphere, without changing their hydrodynamic characteristics and zeta potential, which determine the possibility of effective internalization into the cell.

## Figures and Tables

**Table 1:** The effect of irradiation conditions on the intrinsic viscosity of FA-PVP-C_60_ and PVP solutions.

Sample	Irradiation Atmosphere	Dose, kGy	[η]*, dL/g	${\bar{M}}_{v}$*, kDa
FA-PVP-C_60_	Unirradiated		0.254 ± 0.015	-
	Air	3	0.180 ± 0.017	
		8	0.313 ± 0.016	
	Xenon	3	0.244 ± 0.008	
		8	0.202 ± 0.005	
PVP	Unirradiated		0.190 ± 0.008	47 ± 2
	Air	3	0.171 ± 0.005	32 ± 2
		8	0.332 ± 0.044	108 ± 14
	Xenon	3	0.249 ± 0.038	-
		8	0.233 ± 0.008	-

* Data present as mean ± S.D.

## Data Availability

Data are contained within the article.
